# Morphological Expression of *KIT* Positive Interstitial Cells of Cajal in Human Bladder

**DOI:** 10.1016/j.juro.2010.03.005

**Published:** 2010-07

**Authors:** Louise Johnston, Siobhan Woolsey, Rebecca M.J. Cunningham, Hugh O'Kane, Brian Duggan, Patrick Keane, Karen D. McCloskey

**Affiliations:** Centre for Cancer Research and Cell Biology, School of Medicine, Dentistry and Biomedical Sciences, Queen's University Belfast and Department of Urology, Belfast City Hospital (SW, HOK, BD, PK), Belfast, United Kingdom

**Keywords:** urinary bladder, interstitial cells of Cajal, muscle, smooth, proto-oncogene proteins c-kit, anatomy and histology, ATP, adenosine triphosphate, ICC, interstitial cell of Cajal, LP, lamina propria, PBS, phosphate buffered saline, PICC, perivascular ICC, rER, rough endoplasmic reticulum, SM, smooth muscle, SMC, SM cell, TEM, transmission electron microscopy, vAChT, vesicular acetylcholine transferase

## Abstract

**Purpose:**

We investigated the 3-dimensional morphological arrangement of *KIT* positive interstitial cells of Cajal in the human bladder and explored their structural interactions with neighboring cells.

**Materials and Methods:**

Human bladder biopsy samples were prepared for immunohistochemistry/confocal or transmission electron microscopy.

**Results:**

Whole mount, flat sheet preparations labeled with anti-*KIT* (Merck, Darmstadt, Germany) contained several immunopositive interstitial cell of Cajal populations. A network of stellate interstitial cells of Cajal in the lamina propria made structural connections with a cholinergic nerve plexus. Vimentin positive cells of several morphologies were present in the lamina propria, presumably including fibroblasts, interstitial cells of Cajal and other cells of mesenchymal origin. Microvessels were abundant in this region and branched, elongated *KIT* positive interstitial cells of Cajal were found discretely along the vessel axis with each perivascular interstitial cell of Cajal associated with at least 6 vascular smooth muscle cells. Detrusor interstitial cells of Cajal were spindle-shaped, branched cells tracking the smooth muscle bundles, closely associated with smooth muscle cells and vesicular acetylcholine transferase nerves. Rounded, nonbranched *KIT* positive cells were more numerous in the lamina propria than in the detrusor and were immunopositive for anti-mast cell tryptase. Transmission electron microscopy revealed cells with the ultrastructural characteristics of interstitial cells of Cajal throughout the human bladder wall.

**Conclusions:**

The human bladder contains a network of *KIT* positive interstitial cells of Cajal in the lamina propria, which make frequent connections with a cholinergic nerve plexus. Novel perivascular interstitial cells of Cajal were discovered close to vascular smooth muscle cells, suggesting interstitial cells of Cajal-vascular coupling in the bladder. *KIT* positive detrusor interstitial cells of Cajal tracked smooth muscle bundles and were associated with nerves, perhaps showing a functional tri-unit controlling bladder contractility.

First described by Ramón y Cajal,^1^ ICCs exist in SM tissues, including genitourinary tract regions, where they are thought to modulate SM activity.^2^ Current interest in bladder ICCs was augmented by reports of their putative association with bladder pathophysiology with ICC populations up-regulated in obstructed guinea pig bladders^3^ and overactive bladder cases,^4^ suggesting that ICC over expression may generate aberrant contractility.

Convincing evidence shows that ICCs are present in normal bladders and have physiological profiles consistent with modulatory functions. Initial observations of cells morphologically resembling ICCs in guinea pig and human bladders were based on cyclic guanosine monophosphate and vimentin immunohistochemistry.^5^ ICCs were later identified in guinea pig bladders using the established ICC marker anti-*KIT*.^6^ This was since confirmed in guinea pig,^4^, ^7^ mouse^8^ and human^4^, ^9^ bladders.

Distinct LP and detrusor ICC subpopulations are found in guinea pig and murine bladder walls.^10^, ^11^ Stellate *KIT* positive LP-ICCs form loose networks interconnected by gap junctions and make structural associations with nerves.^10^, ^11^ Detrusor ICCs lie axial along the edge of SM bundles, closely associated with nerves, suggesting that ICCs, SMCs and nerves may form functional tri-units.^8^, ^10^ LP and detrusor ICCs show spontaneous electrical and Ca^2+^ signaling,^7^, ^12^, ^13^, ^14^ respond to exogenous application of neurotransmitters such as carbachol or adenosine triphosphate,^13^, ^15^ and express purinergic and cholinergic membrane receptors.^16^, ^17^

Most studies of bladder ICCs have been done in animal models but *KIT* expression was noted in human bladder frozen or paraffin sections. However, to our knowledge the 3-dimensional profile of ICCs in the human bladder wall, their morphological properties or their relationships with neighboring cells have not yet been characterized. We investigated the morphological arrangement of *KIT* positive ICCs in the human bladder wall using whole mount preparations and confocal microscopy, and examined their interactions with nerves and SM. We also studied ICC ultrastructural characteristics by TEM.

## Materials and Methods

### Tissue Samples

Transurethral cold cup biopsies were obtained from 70 patients undergoing oncological clinical investigation who provided informed written consent. Of this sample population we used 49 preparations and the remainder were used in a separate study. Biopsies were taken remote from the clinical interest site, comprising urothelium, LP and rarely some SM from underlying detrusor. A sample of normal full-thickness bladder was obtained from a patient undergoing reconstructive surgery. There were no reports of abnormal voiding in the patients and, thus, samples were considered to be from control/normal bladders. Ethical approval was granted by the Office of the Research Ethics Committee Northern Ireland, United Kingdom, in accordance with the Declaration of Helsinki and Good Clinical Practice.

### Immunohistochemistry

Tissues were processed for immunohistochemical analysis as described previously.^6^ Samples were fixed in 4% paraformaldehyde or acetone, washed in PBS, blocked and permeabilized in PBS containing 1% bovine serum albumin and 0.05% Triton X-100, and incubated in primary antibodies for 24 hours. Primary antibodies were raised against human immunogens, including anti-*KIT* (1:200), anti-vimentin (1:200), anti-SM myosin (1:200) (Sigma®), anti-vAChT (Santa Cruz Biotechnology, Santa Cruz, California) (1:2,000) and anti-mast cell tryptase (Abcam®) (1:200). After washing to remove unbound antibody tissues were incubated with the secondary antibodies Alexa Fluor® 488 and 594 (1:200) for 1 hour, washed and mounted on slides with glass coverslips. In experiments to visualize SM by F-actin staining tissue was incubated with phalloidin-tetramethylrhodamine isothiocyanate (Sigma) for 24 hours and washed in PBS.

Control samples were prepared by omitting antibodies to assess tissue autofluorescence. Primary antibody omission to test secondary antibody specificity and guinea pig bladder served as a positive control.

### Microscopy

#### Confocal

Slides were imaged with a C1 confocal imaging system mounted on an upright Eclipse 90i microscope (Nikon, Tokyo, Japan). Fluorophores were excited sequentially with a 405 nm laser diode, and an argon (488 nm) or HeNe (543 nm) laser. Resulting emissions were collected through appropriate filters to photomultiplier tubes. Images were acquired using EZ-C1 software (Nikon), analyzed and reconstructed using MetaMorph® and Photoshop®. Cell length was measured from optical section projections and is shown as the mean ± SD.

#### Transmission electron microscopy

Tissue from the full-thickness human bladder specimens was fixed in Karnofsky fixative (2.5% glutaraldehyde and 2% paraformaldehyde) and processed for TEM. After washing in sodium cacodylate buffer, post-fixation in osmium tetroxide and dehydration samples were infiltrated with epoxy resin (TAAB Laboratories Equipment, Aldermaston, United Kingdom) and embedded in BEEM® capsules. Blocks were sectioned with an UltraCut® ultramicrotome. Regions of interest were identified in 0.5 μm semi-thin sections stained with toluidine blue. Sections (70 nm) were collected to copper grids and stained with uranyl acetate and lead citrate before viewing with a 100CXII TEM (Jeol®).

## Results

### Lamina Propria ICCs

**Figure 1:**
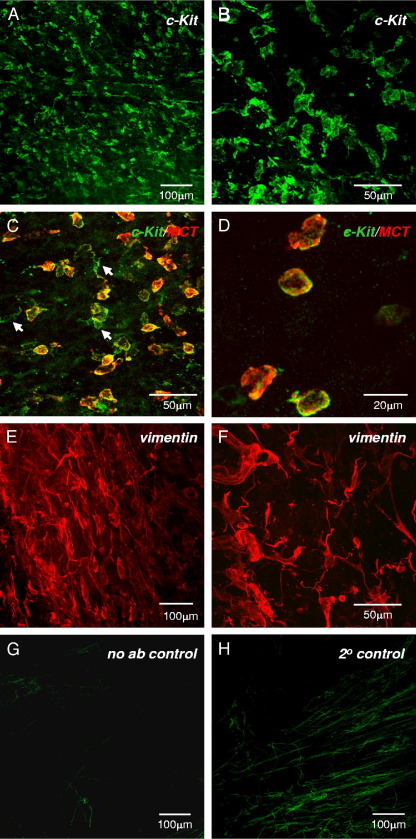
Human bladder LP-ICCs. *A*, whole mount preparation of human bladder muscosa labeled with anti-*KIT* shows *KIT* positive cell network. *B*, higher magnification shows *KIT* positive cell stellate or branched morphology and interconnections. *C* and *D*, *KIT* (green areas) and mast cell tryptase (*MCT*) (red areas) antibody double labeling reveals small, rounded mast cells (orange areas). Arrows indicate *KIT* positive, mast cell tryptase negative ICCs with stellate morphology. *E* and *F*, typical micrographs of vimentin positive LP-ICCs show morphological range, indicating heterogeneous cell population. *G*, representative autofluorescence control imaged with similar settings shows minimal autofluorescence confined to collagen/elastin-type fibers. *H*, representative low level fluorescence from secondary only control, imaged in similar fashion.

Whole mount preparations labeled with *KIT* antibodies revealed remarkable expression of *KIT* positive cells in the human bladder wall in 22 patient samples. An advantage of whole mount preparations over thin cryo/paraffin sections is the ability to investigate 3-dimensional cellular arrangements by acquiring z-series of optical sections. LP *KIT* positive cells were numerous and stellate with several lateral branches from the cell body (fig. 1,*A*). They resembled guinea pig bladder *KIT* positive LP-ICCs.^10^ Many made associations with each other to form a loose network (fig. 1, *B*). Mean LP-ICC length was 53.1 ± 13.1 μm on 74 measurements. Rounded, nonbranched *KIT* positive cells, typical of mast cells (fig. 1, *C* and *D*), were clearly distinct from LP-ICCs (mean length 14.9 ± 3.5 μm on 123 measurements). Anti-*KIT* and anti-mast cell tryptase double labeling resulted in co-labeling of cells with the rounded morphology but not of stellate cells, which were only *KIT* positive, substantiating the notion that the former were mast cells.

Four patient samples labeled with anti-vimentin revealed abundant LP immunopositive cells. Vimentin positive cells had a range of morphologies, including bipolar, rounded and stellate shapes, consistent with a heterogeneous cell population (fig. 1, *E* and *F*). They formed interconnected networks. We determined whether a proportion of the network was also *KIT* positive but double labeling experiments with *KIT* and vimentin antibodies were largely unsuccessful due to the different fixation protocols required for each antibody, ie acetone for *KIT* and paraformaldehyde for vimentin. The limited supply of biopsies precluded development of a protocol to reliably label *KIT* and vimentin in the same tissue but this should be feasible in the future.

Control experiments were done to assess tissue autofluorescent properties. Samples were imaged using the same imaging parameters as experimental tissues. Background tissue autofluorescence was minimal and limited to areas of numerous structures resembling collagen/elastin fibers (fig. 1, *G*). Secondary-only controls to assess secondary antibody specificity were processed and imaged as described. Again, low level fluorescence was noted in fibers (fig. 1, *H*). In most samples background fluorescence did not present significant difficulties when imaging regions of interest.

### ICC-LPs and Cholinergic Nerves

**Figure 2:**
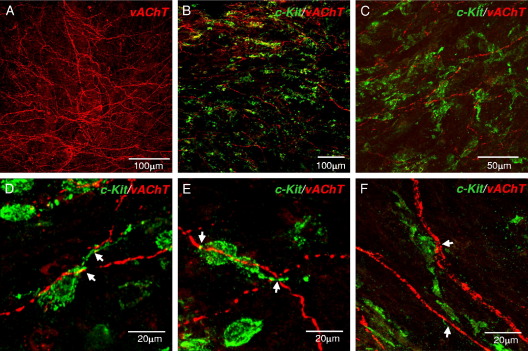
ICCs and mucosal cholinergic nerves. *A*, cholinergic nerve plexus in human bladder mucosa labeled with anti-vAChT. *B* and *C*, low magnification of preparations co-labeled with anti-*KIT* (green areas) and anti-vAChT (red areas) show ICC network-cholinergic plexus associations. *D* to *F*, high magnification reveals ICCs and cholinergic nerves with points of close association (arrows).

Human bladder LP contains a rich cholinergic plexus, as shown by anti-vAChT labeling in 8 patient samples (fig. 2,*A*). This cholinergic plexus made frequent points of close association with the LP-ICC network in 4 patient samples co-labeled with anti-*KIT* (fig. 2, *B* to *F*). ICCs were often associated with more than 1 cholinergic fiber (fig. 2, *D* to *F*), indicating the potential existence of an efficient communication system between ICCs and submucosal nerves.

### ICCs and Microvasculature

**Figure 3:**
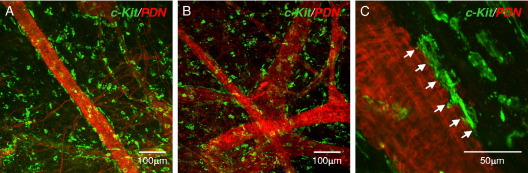
ICCs and bladder microvessels. *A* and *B*, bladder wall microvessels labeled with phalloidin (*PDN*) (red areas) were associated with abundant *KIT* positive ICCs (green areas). *C*, representative higher magnification micrograph shows PICC elongated, branched morphology and connections to at least 6 vascular SMCs (arrows).

The bladder LP region has an extensive microvasculature and most biopsies examined had visible blood vessels (fig. 3). Small arterioles and venules formed anastomosing networks, as revealed by labeling with phalloidin-tetramethylrhodamine isothiocyanate, which binds to filamentous actin. Phalloidin and anti-*KIT* double labeling in 2 patient samples showed associations between ICCs and microvessels in which *KIT* positive cells ran in parallel with the vessel long axis, positioned discretely along the vessel. These PICCs were associated with the outer vessel surface. At high magnification branches of a single ICC were close to at least 6 vascular SMCs (fig. 3, *C*).

### *KIT* Positive Cells in Detrusor

**Figure 4:**
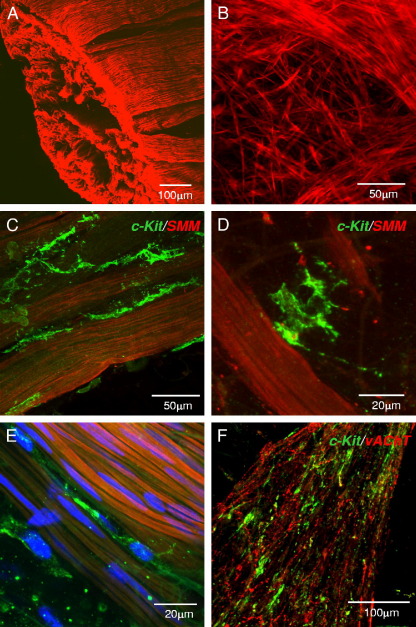
Human detrusor ICCs. *A*, human detrusor labeled with anti-SM myosin (*SMM*). *B*, at mucosa-detrusor interface SMCs were arranged in loose network, which was organized into distinct bundles in detrusor muscularis. *C* to *E*, representative human detrusor co-labeled with anti-SM myosin and anti-*KIT* (green areas). Elongated, branched *KIT* positive ICC track SM bundles and occupy spaces between bundles with obvious spiky morphology. *E*, nuclei counterstained with 4,6-diamidino-2-phenylindole (blue areas). *F*, detrusor ICCs (green areas) associated with vAChT nerves (red areas).

Detrusor cells expressing *KIT* were studied in biopsies that included some underlying detrusor and the full-thickness specimen of normal bladder. Anti-SM myosin labeling showed fine SMC networks at the mucosa-detrusor interface, which were increasingly organized into characteristic bundles as we imaged deeper into the detrusor (fig. 4,*A* and *B*). Anti-*KIT* co-labeling in 4 patient samples showed *KIT* positive ICCs on the boundary of SM bundles, positioned discretely along the longitudinal bundle axes. These ICCs had an spindle, bipolar morphology but, unlike SMCs, had lateral branches, giving a spiky appearance (fig. 4, *C* to *E*). They did not form interconnected networks but some ICCs made connections with each other (fig. 4, *C*). A smaller proportion of detrusor ICCs were more stellate and typically found in the interstitial spaces between SM bundles (fig. 4, *D*). Rounded *KIT* positive cells resembling mast cells were also present but notably less numerous than in LP. Detrusor ICCs were also associated with vAChT labeled cholinergic nerve fibers (fig. 4, *F*).

### Electron Microscopy

**Figure 5:**
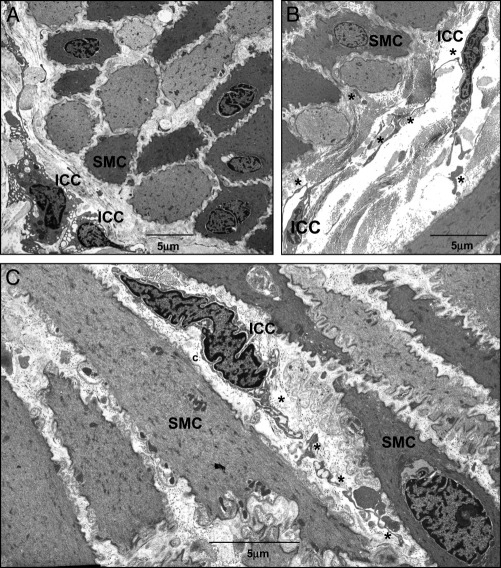
*A* to *C*, representative TEM images of human bladder reveal abundant SMCs and ICCs. *C*, asterisks indicate branched ICC projections. *c*, collagen.

**Figure 6:**
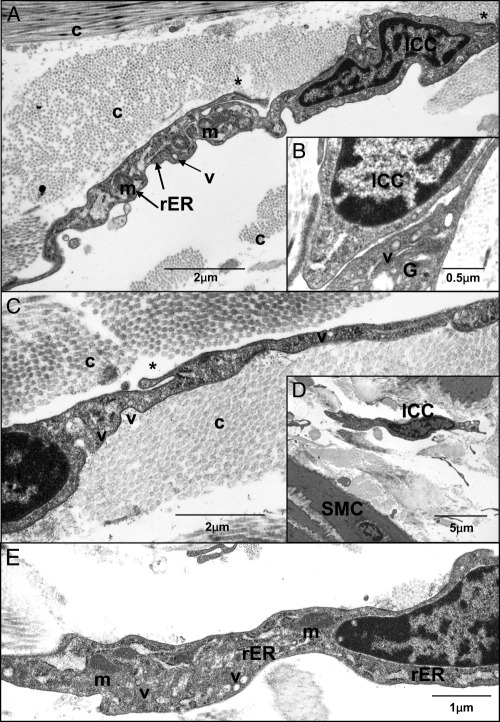
*A* to *E*, typical human bladder ICC ultrastructural characteristics include mitochondria (*m*), Golgi (*G*), vesicles (*v*), rER and branched projections (asterisks). Abundant collagen (*c*) was present in interstitial spaces. In contrast to SMCs, ICCs did not contain thick filaments, dense bands or dense bodies and had incomplete basal lamina. *B*, close association between 2 ICCs. *E*, higher magnification of *D*.

TEM of human bladder samples showed cells with ICC ultrastructural characteristics. Figure 5 shows representative micrographs in which ICCs were distinct from SMCs in location, morphology and ultrastructure. On transverse section ICCs were located on the edge of SM bundles and on longitudinal section ICCs were oriented in parallel with SMCs (fig. 5). LP-ICCs were found in the connective tissue matrix, where collagen fibers were abundant (fig. 6). ICCs were highly branched and appeared to make contact with each other (figs. 5, *B* and 6, *B*), consistent with mentioned observations. They did not contain dense bodies or dense bands typical of SMCs and thick filaments were not observed but they had mitochondria, a discontinuous basal lamina, free ribosomes, numerous vesicles, Golgi and a well developed but not dilated rER. Membrane specializations such as caveolae, which were abundant in SMCs, were not found in ICCs but we noted examples of membrane fused vesicles in several ICCs (fig. 6).

## Discussion

We noted the expression of *KIT* positive ICCs in the human bladder wall, and examined morphological interactions between ICCs and other intramural cells. ICCs but not SMCs or fibroblasts express *KIT* protein,^18^ a receptor tyrosine kinase encoded by the proto-oncogene *c-Kit*. We previously used anti-*KIT* to positively identify ICCs in guinea pig and mouse bladders.^6^, ^8^ In the current study we focused on the human bladder. Our experimental model of antibody labeling whole mount flat sheets and confocal optical sectioning enabled study of the 3-dimensional arrangement of bladder ICCs and their associations with nerves, and vascular and detrusor SM. Thus, this work advances initial reports of *KIT* expression in frozen or paraffin human bladder sections in which it was not feasible to record details of cell morphology or cellular interactions.^9^, ^19^, ^20^

### Lamina Propria ICCs

The complex bladder mucosa comprises urothelium, nerves, blood vessels, collagen and elastin fibers, mast cells and ICCs and, while classically considered a passive barrier, it is now known to release many factors in response to the local environment or to bladder wall distention/mechanical stretch due to the urine volume accommodated.^21^ These factors include adenosine triphosphate,^22^ acetylcholine and nitric oxide, and are thought to signal to neighboring mucosal nerves, ICCs or neighboring urothelial cells. We previously proposed that in guinea pig bladders LP-ICCs act as a conduit relaying information from urothelium to detrusor.^10^ Others share this view and suggested that LP-ICCs transduce, amplify or integrate signals in the bladder wall.^23^

We found a loose network of stellate, *KIT* positive ICCs just below the urothelium, extending to the detrusor. This observation correlates with the findings of Sui et al, who noted suburothelial interstitial cells connected via Cx43 gap junctions on immunohistochemistry and TEM.^11^ Our finding that the *KIT* positive LP-ICC network is associated with a cholinergic plexus indicates that LP-ICCs could be modulated by parasympathetic nerve inputs, consistent with a study that ICCs in this region express muscarinic M_3_ receptors.^17^ However, this hypothesis needs further study since it was reported that isolated LP-ICCs do not respond to cholinergic stimulation by firing Ca^2+^ transients.^15^ Our findings may also support the hypothesis that LP-ICCs participate in a sensory transduction system.^12^, ^23^ While the precise mechanisms have not yet been elucidated, LP-ICCs and the mucosal cholinergic plexus may work together to sense and respond to local signals released from urothelium.

### Perivascular ICCs

The novel finding of PICCs on bladder microvessels presents the intriguing possibility that ICC-vascular coupling exists in the bladder. Similar cells were identified in the gallbladder^24^ but to our knowledge the physiological significance of such PICCs in these organs has not yet been explained. The scenario is reminiscent of neurovascular coupling in the brain, in which contact between astrocytes and arteriolar SM is thought to regulate blood flow and match it to metabolic needs.^25^ Bladder wall vascular perfusion is a key determinant of normal bladder contractility since in vivo ischemia in animal models induces bladder overactivity.^26^ PICCs may provide an elegant mechanism of sensing bladder metabolic needs and subsequently modulating local perfusion.

### Detrusor ICCs

Human bladder detrusor ICCs are similar to their guinea pig and mouse counterparts in morphology, arrangement and relationships with detrusor SM.^8^, ^10^ The physiological roles of detrusor ICCs have not been fully determined from studies in animal models but they may modulate SM activity. Fluorescent Ca^2+^ imaging of bladder sheets showed ICCs and SMCs firing spontaneous Ca^2+^ transients at distinctive frequencies.^7^, ^13^ It was suggested that ICCs may control the minimum firing frequency of SMCs or act as multiple pacemakers, providing input to adjacent SMCs.

ICCs are clearly present in normal tissue and presumably are maintained because they have specific roles in bladder function. However, the ICC role may be more apparent in pathophysiological conditions. Biers et al noted increased *KIT* positive ICC expression in overactive human bladder samples compared with normal samples.^4^ Kubota et al reported an increased ICC population in the guinea pig bladder after outlet obstruction.^3^ The tyrosine kinase blocker imatinib mesylate decreased spontaneous electrical and mechanical activity in human and guinea pig bladder strips, and improved capacity and compliance in animal cystometry experiments.^4^, ^27^, ^28^ Imatinib mesylate was more effective in overactive bladders from spinal cord transected animals than from controls,^29^ indicating that overactivity may be linked to increased *KIT* positive ICC expression or *KIT* signaling pathway activation.

Ultrastructural examination of putative ICCs by TEM remains one of the most reliable means of identifying ICCs in a preparation. In agreement with the recent study by Rasmussen et al^30^ our series shows that cells with the ICC ultrastructural profile at sites identified by *KIT* immunohistochemistry are present in the human bladder wall. Human bladder ICCs share similarities with other ICCs, including absent thick filaments, dense bodies or dense bands typical of SMCs, and mitochondria, ribosomes, vesicles, Golgi and a well developed nondilated rER. Membrane caveolae typically found in SMCs were not present in ICCs. Absence of a fibronexus, which is a defining characteristic of myofibroblasts, was consistent with other published human bladder TEM micrographs,^11^, ^30^ indicating that these cells are unlikely to have a myofibroblastic phenotype. Overall our TEM findings and those in the mentioned reports seem to suggest that human bladder ICCs more closely fit the profile of the ICC family phenotype.

## Conclusions

Our findings show the distinctive morphological arrangements of *KIT* positive ICCs in the LP and detrusor regions of the human bladder wall. ICCs made structural interactions with cholinergic nerves in each region and were closely associated with detrusor SM. To our knowledge we report a previously unknown ICC subtype associated with microvessels and we examined human bladder ICC ultrastructural properties. These novel findings reveal the extent of *KIT* positive ICC expression in the human bladder and provide an essential foundation for future studies in the human bladder.
